# Pandemics and technology engagement: New evidence from m‐Health intervention during COVID‐19 in India

**DOI:** 10.1111/rode.12909

**Published:** 2022-07-12

**Authors:** Sawan Rathi, Anindya S. Chakrabarti, Chirantan Chatterjee, Aparna Hegde

**Affiliations:** ^1^ Indian Institute of Management Ahmedabad Gujarat India; ^2^ Economics Area, Indian Institute of Management Ahmedabad Gujarat India; ^3^ Science Policy Research Unit University of Sussex Business School Brighton UK; ^4^ ARMMAN and Cama Hospital Mumbai India

**Keywords:** gender, information acquisition, information provision, m‐Health, pandemic, technology engagement

## Abstract

Information provision for social welfare via cheap technological media is now a widely available tool used by policymakers. Often, however, an ample supply of information does not translate into high consumption of information due to various frictions in demand, possibly stemming from the pecuniary and non‐pecuniary cost of engagement, along with institutional factors. We test this hypothesis in the Indian context using a unique data set comprising 2 million call records of enrolled users of ARMMAN, a Mumbai‐based nongovernmental organization that sends timely informational calls to mobile phones of less‐privileged pregnant women. The strict lockdown induced by COVID‐19 in India was an unexpected shock on engagement with m‐Health technology, in terms of both reductions in market wages and increased time availability at home. Using a difference‐in‐differences design on unique calls tracked at the user‐time level with fine‐grained time‐stamps on calls, we find that during the lockdown period, the call durations increased by 1.53 percentage points. However, technology engagement behavior exhibited demographic heterogeneity increasing relatively after the lockdown for women who had to borrow the phones vis‐à‐vis phone owners, for those enrolled in direct outreach programs vis‐à‐vis self‐registered women, and for those who belonged to the low‐income group vis‐à‐vis high‐income group. These findings are robust with coarsened exact matching and with a placebo test for a 2017–2018 sample. Our results have policy implications around demand‐side frictions for technology engagement in developing economies and maternal health.

## 1 INTRODUCTION

The COVID‐19 pandemic has renewed interest in information provisioning in both academic and policy discourse. In recent times, the cost for information provisioning has gone down drastically (Aker, 2010; Aker & Mbiti, 2010), and widespread reach due to a variety of digital media has substantially reduced the cost of dissemination (Goldfarb & Tucker, 2019). As policymakers observed the ease of access to low‐cost digital technology for the general populace, there has been a push toward information dissemination by utilizing communication media on a mass scale. Such intervention allows social planners to address information asymmetry arising from a digital divide between *information‐rich* and *information‐poor* people (Ramsetty & Adams, 2020; Watts, 2020).

However, while the supply of information expanded, the demand side of information acquisition behavior is not well understood. There could be scenarios where despite information being disseminated, the actual consumption of information falls short, leading to the failure of the intended cause of the focal intervention. A prominent example of such intervention comes from the recent experience of the Indian government introducing prerecorded messages that are heard by the callers making a phone call within India after the COVID‐19 pandemic began. The effect of this intervention is not known clearly. Sadish et al. (2021), however, observed that till a quarter into the pandemic, misinformation was quite prevalent. In particular, they show that prerecorded messages lead to less engagement and less reduction in misinformation than direct phone calls.

While active phone calls can lead to more engagement, such a policy has two problems for mass‐scale intervention. First, it can be prohibitively expensive to implement on a large scale. Second, it is not well understood which factors affect such engagement. Two mechanisms may drive the phenomenon of low engagement. First, access to information itself might be low, for example, due to a lack of resources to procure a communication device. Second, although people may have access to information for all practical purposes, they may fail to utilize the information due to frictions unobserved by the supplier of information. A recent example is the failure of global uptake of contact tracing or vaccine registration apps (Ivers & Weitzner, 2020), although they have been made available to the populace.

The goal of this paper is to disaggregate and investigate sources of friction in technology engagement. We examine this question using a unique data repository of the mMitra program of a Mumbai‐based nonprofit organization named Advancing Reduction in Mortality and Morbidity of Mothers, Children, and Neonates (ARMMAN). The data set comprises 2 million call records of mobile phone–based healthcare (m‐Health, hereafter) interventions on less‐privileged pregnant women in India to improve maternal health outcomes.

The registered women receive 141 individualized prerecorded messages via voice calls throughout pregnancy until the child is 1 year old, each prerecorded message lasting 60–120 s.^1^ During the pre‐lockdown period, the prospects of these women listening to the complete prerecorded voice message or even picking up the phone at times would be relatively low, owing to their family and work responsibilities. However, the cost was likely reduced during the lockdown for two reasons. One, the loss of jobs and wages during the pandemic time reduces the opportunity cost. Two, the general level of activity would likely go down during the lockdown phase due to the strict nature^2^ of the lockdown imposed with the pecuniary cost of breaching the lockdown guidelines, including going out of the home.

**FIGURE 1:**
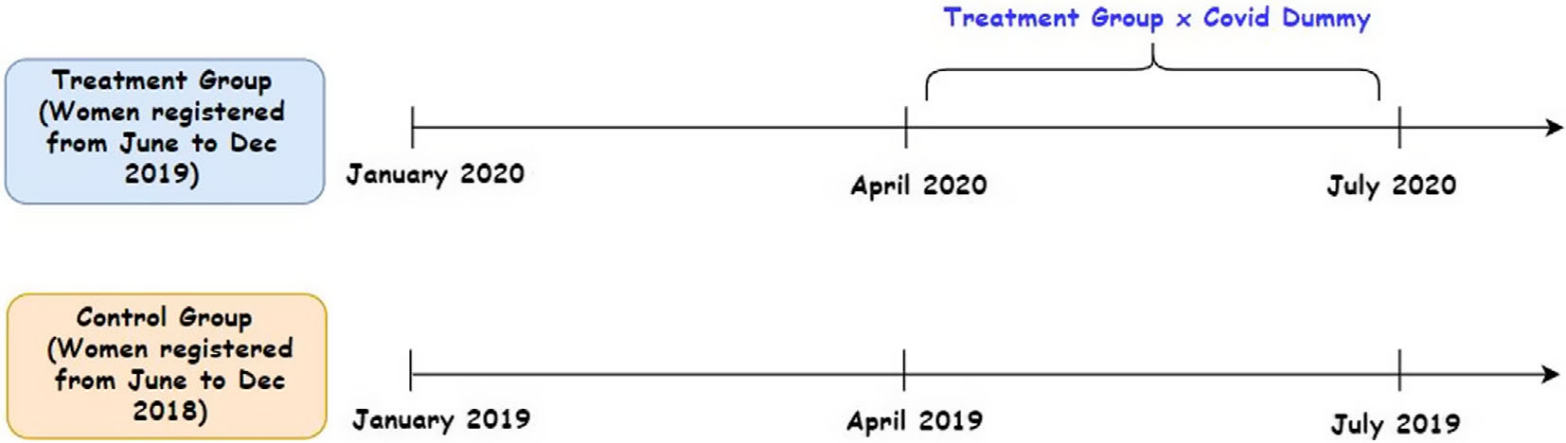
Timeline of treatment and control groups. Call details of the treatment group are studied from January 2020 to July 2020 and of the control group from January 2019 to July 2019. COVID dummy indicates months from April

We capture this differential technology engagement in our empirical analysis by applying a difference‐in‐differences methodology using COVID‐19 as an exogenous shock. We design our treatment group around the women who registered with the ARMMAN between June 2019 and December 2019 and our control group around the women who registered between June 2018 and December 2018. We keep track of the prerecorded voice messages they received from January to July in 2020 and 2019, respectively. The rationale for constructing a treatment group with registration in 2019 is to capture the effect of COVID‐19 on their technology engagement behavior during the period of pregnancy that shall happen in 2020. We choose 3 months before and 4 months after the announcement of lockdown on March 22, 2020, that is, January to March and April to July, as the pre‐and posttreatment periods for both the groups. The posttreatment period represents the period of the strictest lockdown in the country. The control group follows the same timeline to control for seasonal and time trend effects. The timeline in Figure 1 provides a pictorial view of the treatment and control groups.

We hypothesize that technology engagement among the poor is influenced by three factors: *ownership of technology*, *accessible channels of information*, and *availability of technology*.

We capture the ownership of technology using the “phone owner” attribute in the data. When a woman is enrolled in the program, she fills in the details of who is the owner of the phone in the enrollment form. In the data, we find that in most cases the owner is the woman herself or her husband and in a small fraction of cases, a neighbor owns the phone. If the husband is the phone owner, the constraints of accessing the phone for women reduce during the lockdown as the husband would be more at home and therefore accessibility increases.

“Accessible channels of information” is captured via the enrollment channel. There are two routes through which women can enroll with ARMMAN. The first is through the hospital vertical in which health workers posted in clinics of municipal/government/private hospitals register women during their first check‐up visit. The second is through the community vertical, which works outside the hospital, especially in the slum areas. These enrollments are done through partner nongovernmental organizations in slum communities. Community health workers (called “Sakhi”‐s) are trained to directly enroll women in the early pregnancy stages for a small incentive, and past work shows their criticality in the Indian public health ecosystem (Kaletski & Prakash 2017). Compared to women who would directly go to the hospital for registration and consult doctors, the quality of information for the women registered via community health workers will be lower. We hypothesize that the women enrolled via community health workers would be more willing to acquire complementary information from ARMMAN.

“Availability of technology” is captured by “income range.” There are seven categories in the income data with the following ranges—Rs. 0–5,000, 5,000–10,000, 10,000–15,000, 15,000–20,000, 20,000–25,000, 25,000–30,000, and above 30,000 per month. Upon registration, the women have been categorized accordingly, and we do not see the exact income values. The cutoff of Rs. 10,000 splits the sample into two almost equal halves, 53% and 47%, to be exact. The mean income for the poorer half is approximately Rs. 6,500 per month and the same for the richer half is approximately Rs. 14,000 per month.^3^ In comparison, cheap phones available in the market would cost around Rs. 4,000–6,000. Also, to keep the service active, one has to pay fees. Anecdotal evidence suggests that in many cases, poor people often discontinue service due to nonpayment of the service fees. This prompts us to hypothesize that with more than a 100% differences in average incomes, the lower‐income group would be less likely to avail technology, leading to lower engagement.

To investigate these hypotheses, we split the population of women from January 2020 to July 2020 according to the three specifications: phone ownership, enrollment channel, and monthly income. We specify a difference‐in‐differences model, taking lockdown as the exogenous shock. Our estimates suggest a significant rise in the *duration of calls*, our key outcome variable of interest, during the lockdown phase for women who do not own a phone, for women enrolled via community channel, and for women belonging to the lower‐income group. Our results are robust with respect to coarsened exact matching (CEM) and a placebo test, which ameliorates concerns about ad hoc choice of controls.

While the first two results of factors influencing technology engagement are in the expected directions, the third result goes against the hypothesis that poorer women would be less likely to engage more with technology due to the lack of availability of the communication device in working conditions. A possible explanation for this result is that conditional on purchasing the phone, the information provision by ARMMAN is the only source of credible and useful information to these low‐income group women, which leads to higher engagement, whereas the comparatively richer group of women have access to complementary sources for information. This explanation highlights the role of complementary information in line with the second mechanism regarding accessible channels of information.

To characterize the behavioral stickiness of these women, we divide the call duration as a percentage of the total call length into three zones of red, amber, and green, following past work by Collins et al. (2014) and Clay‐Williams et al. (2019). Red zone indicates if the call gets heard for less than 33% of the total call length. Amber zone indicates the duration range between 33% and 66%, and green zone indicates if the call gets heard for more than 66% of the total call length. The difference‐in‐differences estimates reveal an increase in green zone calls and decrease in amber and red zone calls, with a higher magnitude for the amber zone. Thus, the switch occurs predominantly from the amber zone to the green zone.

Our work has important policy implications. Engaging with technology, particularly m‐Health interventions and their associated behavioral channels and mechanisms, has been gaining recent attention in the broader technology diffusion literature (Geels et al., 2021; Gillingham & Bollinger, 2021). Our study also directly connects with past work in development economics that demonstrates how people learn and adopt technology (e.g., Sadish et al., 2021; Burkitbayeva et al., 2020; Conley & Udry, 2010; Bandiera & Rasul, 2006, Munshi, 2004, among others). More recent work also shows how mobile phones are welfare‐enhancing broadly and more so for connectivity in healthcare (e.g., Breza et al., 2021; Ghose et al., 2021; Siddique et al., 2021; Banerjee et al. 2020; Ruton et al., 2018; McBride et al., 2018; Ybarra et al., 2014).

Prior work seems negligent, however, in examining sustained engagement and interaction by people post‐adoption at the intensive margin of technology engagement by users. While influential evidence now exists on the welfare‐enhancing role of mobile phones on the extensive margin (Jensen, 2007), the decomposition of the nature of engagement with the technology remains under‐investigated. This is particularly important from a public policy perspective. Recently the United Nations has initiated dialogue among heads of UNGIS^4^ agency members to raise awareness about the importance of unpacking technology engagement and digitization in achieving sustainable development goals (SDGs),^5^ and economists too have joined these conversations (Banerjee et al., 2020; Siddiqui et al. 2020).

Our study informs these conversations and unpacks how women engage with technology like m‐Health interventions for maternity health management with constraints in the developing world. Our findings suggest that while the pandemic has had an adverse effect, especially on women worldwide, including in India (Rathi & Chatterjee 2022; Bau et al. 2021; Ribarovska et al., 2021; Xue & McMunn, 2021; Yaish et al., 2021; Zamarro & Prados, 2021; Deshpande, 2020; Myers et al., 2020 and Ravindran & Shah, 2020, among many others), in some rare contexts like ours, it may have had a positive welfare effect of an increase in technology engagement. While the lack of data does not allow us to investigate whether it translates into better maternal health outcomes, we hope that, on the margin, it may have given the large body of evidence now on the welfare‐enhancing aspect of mobile phones (Cawley & Ruhm, 2011; Dupas, 2011a).

Finally, maternal mortality in India (also in developed nations like the United States^6^) is also a critical public health issue. Although in recent decades the situation has improved, India still contributes to one‐fifth of the global burden of absolute maternal deaths. Given this grand challenge, we are probably the first to examine the intensive margins of technology engagement with m‐Health interventions for women in the developing world, and thus our findings should have broader global healthcare policy implications.

The rest of the paper proceeds as follows. We review the literature and develop research propositions in Section 2. Next, we provide institutional background in Section 3, followed by a discussion of data and empirical identification strategy in Section 4. We then report our findings in Section 5, followed by robustness checks in Section 6. Section 7 summarizes and concludes. We provide a conceptual framework to explain the findings in Appendix C.

## 2 LITERATURE REVIEW AND RESEARCH PROPOSITIONS

### 2.1 Technology engagement through m‐Health

“Mobile health, or m‐Health, is the utilization of short messaging service (SMS), wireless data transmission, voice calling, and smartphone applications to transmit health‐related information or direct care” (Betjeman et al., 2013). Within this literature, there are multiple parallel strands. The first strand caters to how mobile technology has been useful in improving health workers' output. ARMMAN has a program named Mobile Academy, which is a health training course to empower ASHA^7^ workers with better knowledge and improve their services. Several studies exist that evaluate mobile technology and its relevance herein. For example, Tyagi et al. (2020) studied a mobile phone–based intervention, ThinkTB, focused on improving tuberculosis diagnostic practices and found higher healthcare provider engagement based on individual preferences for instructional content.

Modi et al. (2019) evaluated the effectiveness of a mobile phone– and web‐based application, ImTeCHO,^8^ and found that it helped ASHA workers to provide quality MNCH^9^ services in difficult‐to‐reach areas. Nimmagadda et al. (2019) similarly studied the impact of Common Application Software installed on the smartphones of health workers and found it to be not very effective at a large‐scale ReMiND (reducing maternal and newborn deaths) program, which is another m‐Health application that runs on an open‐source platform introduced as a job aid for ASHA workers (Prinja et al., 2018). In Tanzania, the m‐Health for safer deliveries program supported community health workers who used a phone with a user‐friendly decision‐support application so that they could register, counsel, record, screen, and text pregnant women (Battle et al., 2015).

m‐Health literature also focused in the past on how text messages can help consumers change their health behaviors. Project Masiluleke in South Africa (Canales, 2011), Text to Change in Uganda (Chib et al., 2012), Text2Teach in the Philippines (Roble, 2018), and Stop my smoking (SMS) in the USA (Scott‐Sheldon et al., 2016; Ybarra et al., 2014; Carpenter & Cook, 2008) are several studies that measure the impact of text messages on health behavior. Studies specifically focusing on MNCH are RapidSMS in Rwanda (Ruton et al., 2018), Text4Baby in the USA and Russia (Evans et al., 2012), mMom in Vietnam (McBride et al., 2018), and a pilot impact study in rural Guatemala (Prieto et al., 2017). Our study is, however, different from this literature as we focus on the effect of voice‐based messages (a relatively recent upgrade in this intervention) instead of text‐based messages. In addition, our study relates to new work on mobile health platforms and apps that examine the health outcome effects of wearable digital devices, apps, and social media tools (Ghose et al., 2021).

The other strand of m‐Health literature relevant to our study concerns the usage and effectiveness of m‐Health measures in developing countries. Analysis of the efficiency of healthcare organizations and instruments is a complex task (Hollingsworth & Street, 2006). There are different perspectives on how potent m‐Health is used for developing worlds. Betjeman et al. (2013) believe that m‐Health can improve and reduce costs, especially in rural areas. Countering this perspective, Eckman et al. (2016) argue that most m‐Health projects are generic and thus less beneficial for individual needs. Ruton et al. (2018) suggest that m‐Health should not be standalone but considered part of a more comprehensive intervention package to build up health system capacity. Gleason (2015), in addition, suggests that security, cost, interoperability, scalability, and lack of local knowledge are barriers to the use and development of m‐Health initiatives. Given the mixed findings, understanding a new intervention like the one run by ARMMAN is even more crucial, that too its performance during the current pandemic. Further in line with these studies, ARMMAN also faces various technological challenges like the quality of users' phones, availability of electricity supply for charging phones, and frequent changes in mobile phone SIM numbers by members, but how they translate into actual technological engagement should have broader digital policy implications beyond ARMMAN.

### 2.2 Information dissemination in healthcare

A critical aspect of such digital policy, as we describe earlier, is information dissemination, particularly in healthcare contexts. Breza et al. (2021) study how information dissemination via Facebook advertising campaigns about COVID‐19 changes user decisions to stay at home during festive seasons. They find that such interventions are indeed useful. Barili et al. (2021) examine how information transmission between pregnant women shapes the choice of their delivery method. Prior work has also studied how information dissemination impacts health behaviors, resulting in favorable health outcomes (Cawley & Ruhm, 2011; Dupas, 2011a). Our study is focused on one such information dissemination program of ARMMAN, the mMitra.

Some studies show the impact of information on changing habits of peers. Chatterjee et al. (2018) and Debnath and Jain (2020) study the role of spatial peers and social networks in the diffusion of information and show how it increases the uptake of universal health insurance. Other studies show that information provided through telemedicine centers increases the access and uptake of healthcare programs (Mohanan et al., 2016; Delana et al., 2019). Madajewicz et al. (2007) show that information to households that their well water has an unsafe concentration of arsenic increased the likelihood of switching to a safer well.

Dupas (2011a) finds that adolescent girls change their sexual behavior in response to information on the relative risk of contracting HIV by the type of partners. In an older study, Wilson and Chandler (1993) found that 79% of mothers in a village in Lombok, Indonesia, continued to wash their hands with soap 2 years after a 4‐month intervention to promote the practice. Rhee et al. (2005) conducted a randomized controlled trial in Mali in 2003 to find that 49% of households that had received the educational component impregnated their bed nets to fight against malaria, compared with 35% of households that did not. Thomas et al. (1991) have also shown that almost all the impact of maternal education on child health can be explained by indicators of access to information, such as reading the newspaper, watching television, and listening to the radio.

On the contrary, the merits of more information in the health ecosystem are complex (Phelps, 1992) and exist at various margins beyond just access to information. Healthcare is characterized by informational asymmetries between providers and consumers (Amaral‐Garcia et al., 2019). The informational impact depends on the quality and time of information and to whom it is provided (Dupas, 2011b). Our research differs from existing studies in two crucial ways: first, mMitra program of ARMMAN is not a one‐time intervention program. Second, the impact of information dissemination gets studied in the presence of exogenous COVID‐19 health shock, with outcomes studied varying from call duration. This enhances our understanding of technology engagement as a function of the availability of technology, enabling us to unpack more nuanced intensive margins of engagement with technology.

### 2.3 Health shock and behavior

Frank (2004) suggests that in matters related to health, consumers make choices in fear and urgency, trusting the expert; thus, health space can be a fertile ground for behavioral economics researchers. COVID‐19, in our context, has come up as a natural health shock that we can leverage as an experiment to study whether self‐concern in less‐privileged women has promising effects. There are existing studies on assessing the effect of health shocks on community mitigation efforts (Aburto et al., 2010), crisis management and self‐protection (Bennett et al., 2015), natural adoption of protective health behavior (Agüero & Beleche, 2017), and immediate response under the risk of death (Dupas, 2011b). Our study advances this literature, given the grand challenge of maternal mortality in public health settings of developing economies like India, especially when they are hit by an external shock like a pandemic.

### 2.4 Research propositions

Building on the above literature, we test how the lockdown ensuing from COVID‐19 influenced the technology engagement of less‐privileged pregnant women. We measure technology engagement utilizing the call duration, that is, the fraction of the total duration of the calls that the intended recipients listened to. Technology engagement for women is driven by economic or informational constraints. The former constraint may appear if women cannot afford to pick up the call during their regular work time. The latter type of constraints may appear since some women may ignore the information content and not listen to the calls. Building on our literature review, we hypothesize that due to increased time availability during the lockdown, women would spend more time listening to the calls containing prerecorded messages on pregnancy‐related information. We summarize this scenario formally in the following proposition.

Proposition 1 *The duration of calls heard by pregnant women increases during the COVID‐19‐induced lockdown*.

Next, we study how heterogeneity in economic and demographic characteristics influences technology engagement behavior. First, we hypothesize that the women who do not own phones would engage relatively more post lockdown than those who are phone owners. The reasoning here is intuitive. Women who do not own a phone have to borrow the same from their husbands (or neighbors, in some cases) to pick up a call. Since the husband would be working outside the home pre‐pandemic and therefore less reachable, she is less likely to pick up the calls. Therefore, the lack of availability of the phone prevents her from listening to the calls relative to the woman who own the phones herself. However, during the COVID‐19‐induced lockdown, we expect non‐owners to engage relatively more with technology.

Second, women enrolled via community channels would presumably face pecuniary, time availability, or informational constraints that prevent them from going to the hospitals to acquire information. We hypothesize that they would treat informational phone calls as useful and complementary to their existing information set. Therefore, these women should engage relatively more with technology during the lockdown as their constraints become less binding.

Finally, the income level may also influence the technology engagement behavior. We hypothesize that relatively richer households would be more likely to access technology. Conversely, relatively poorer households would be less likely to access technology. Given the range of the income of the households we study, the service cost of mobile phone carriers can often be comparable to the cost of basic necessities like food and medicine. Given that the lockdown also induced a major income shock, we expect such income effects to be more binding for the relatively poorer households.

We summarize these three intuitions in the following proposition:

Proposition 2 *Technology engagement during the COVID‐19‐induced lockdown would be relatively higher for women who are non‐owners, for women accessing information via community workers*, *and for women who belong to the high‐income group*.

In the next section, we lay out our institutional context by describing the firm ARMMAN and its program from where we obtained the data. We also briefly explain the macro‐level effects of the pandemic on India and its healthcare ecosystem.

## 3 INSTITUTIONAL CONTEXT

### 3.1 ARMMAN—An Indian non‐profit organization

ARMMAN is an India‐based nongovernmental and not‐for‐profit organization that was set up to improve the well‐being of pregnant women, new mothers, infants, and children in their early years.^10^ ARMMAN, which stands for “wish” in Hindi, is an acronym for Advancing Reduction in Mortality and Morbidity of Mothers, Children, and Neonates. The goal of ARMMAN is to design and implement interventions that reduce maternal, neonatal, and child morbidity and mortality in less‐privileged urban and rural communities in India by using technology to develop viable interventions and maximize outreach.

This study uses the database of mMitra, one of the first programs started by ARMMAN. The program mMitra, which translates to a *mobile friend*, is a free mobile voice call service for enrolled women to receive critical healthcare information during their pregnancies. Increasing mobile penetration in India positions voice calls as a cheap and fast way to reach women and families. Stage‐based maternal messaging programs like that of mMitra are one of the most successfully scaled programs within the m‐Health domain (Peter et al., 2018). These programs have shown to increase knowledge and utilization of antenatal care services in m‐Health (Watterson et al., 2015).

ARMMAN and BabyCenter^11^ have developed the messages that have been validated by the Federation of Obstetric and Gynaecological Society of India and the National Neonatology Forum. Around 141 individualized voice messages of 60–120 s duration are sent with the following frequency: bi‐weekly during pregnancy, daily for the first week after the child's birth, bi‐weekly again until the third month of infancy, and weekly for month 4 of infancy to month 12. The information transmitted in the calls is matched with the stage of a woman's pregnancy. Calls are sent in the time slot chosen by the women. Women can select to receive the calls in their mother tongue. A trained counselor can be informed about a delivery, abortion, or change in the phone number or time slot.

**FIGURE A1:**
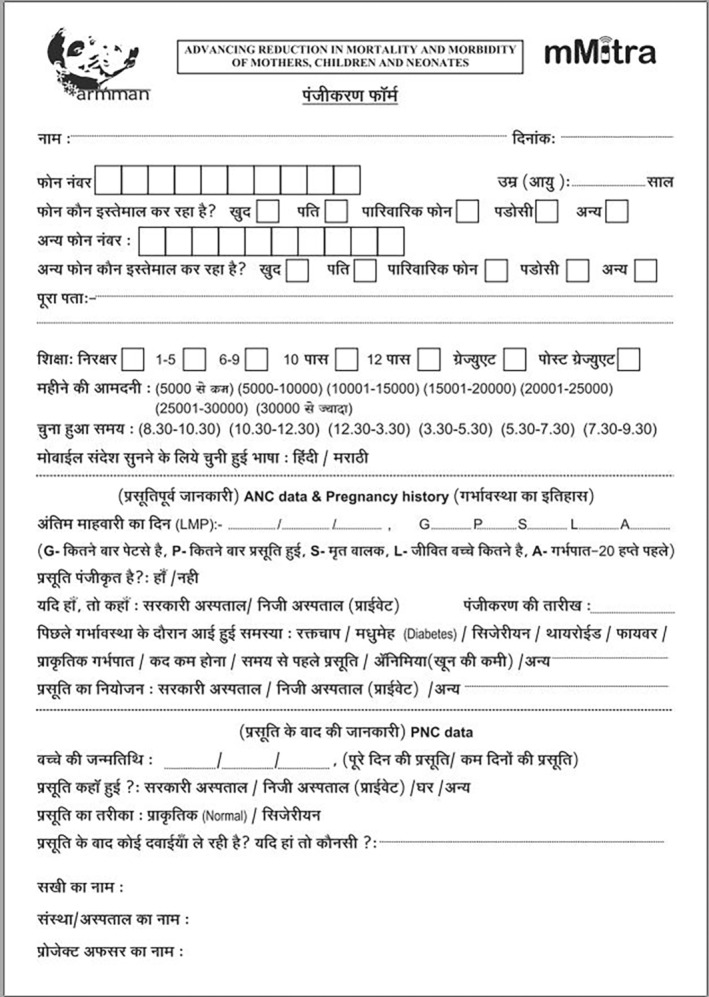
Enrolment form (Hindi). A woman who enrolls with ARMMAN has to compulsorily fill out this enrollment form, which is available in Hindi and Marathi languages. The form captures phone number, phone owner, education, income, call time preference, language preference, pregnancy history, planned place of delivery (private or government hospital), and health issues (if any). *Source*: ARMMAN

**FIGURE A2:**
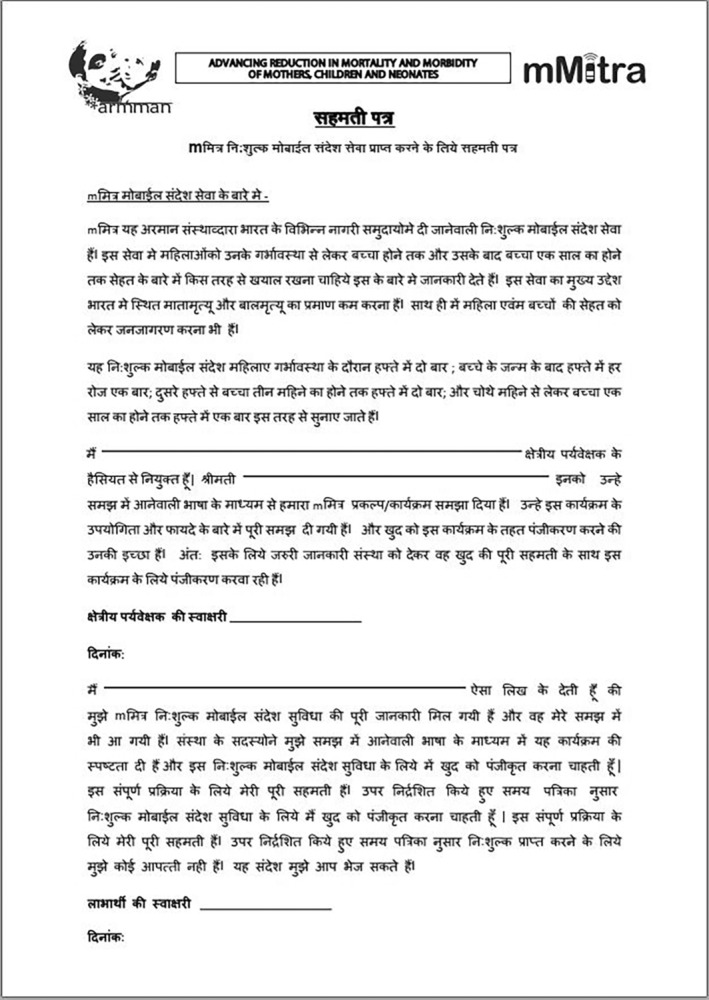
Letter of acceptance. A woman who enrolls in the ARMMAN program has to sign the acceptance letter stating that she has understood the program and its benefits. By signing this letter, she also agrees to receive the informational calls from ARMMAN. *Source*: ARMMAN

To avail the benefits of the mMitra program (which primarily operates in urban slums of Mumbai and vicinity), a woman has to fill out a detailed enrollment form and letter of acceptance (see Figures A1 and A2 in Appendix A). The enrollment form captures her data of mobile phone number, phone owner, education, income range, preference of call time, language preference, pregnancy history, planned place of delivery (private or government hospital), and existing health issues (if any).

### 3.2 COVID‐19 in India

In India, the first case of COVID‐19 was reported on January 30, 2020. On March 4, 2020, 22 new cases were diagnosed. The total number of patients reached 107 by March 15. On March 22, the Government of India announced a strict lockdown. The 68 days of four‐phased‐lockdown started from March 24 to May 31, 2020, to deal with COVID‐19. The lockdown was perhaps required for the world's second‐largest nation with a population of 1.38 billion people. A complete shutdown may have effectively managed the spread of COVID‐19 since India had only seen 131,868 confirmed cases and 3,867 related deaths as of May 24, 2020.^12^ The number of positive cases crossed 10.6 million in January 2021,^13^ and a devastating second wave followed the first wave. Recent work indicates that the effect of COVID‐19 in India shows significant interstate heterogeneity, and the factors explaining this include income, gender, multi‐morbidity, urbanization, lockdown and unlock phases, weather including temperature and rainfall, and the retail price of wheat (Imai et al. 2021).

Except for essential facilities, most of India was closed during the 2020 lockdown.^14^ This episode brought multiple challenges to the already‐overstretched healthcare system. On the one hand, there was the disease burden arising from increasing cases of patients; on the other hand, regular healthcare was disrupted and caused deaths of otherwise high‐risk patients. Less‐privileged people were severely affected by this calamity.^15^ There are limited data on health information, beliefs, and behaviors worldwide that might indicate different exposure to risks from COVID‐19 (Alsan et al., 2020). We use ARMMAN data to examine how during lockdown, women engaged with technology as measured in our earlier‐stated outcome variables.

## 4 DATA AND EMPIRICAL METHODOLOGY

### 4.1 Data

ARMMAN had 2.29 million women enrolled in its mMitra program by 2020. The organization has 40 nonprofit partners and collaborates with 97 hospitals. We got access to ARMMAN's data through one of the coauthors, who is also the founder of ARMMAN. The approval of Institutional Review Board (IRB) to use these data was received from the IRB committee of IIM Ahmedabad, and the approval number is IIMA IRB 2021–11. Our baseline sample comprises demographic and call details of 116,449 women registered (identities were anonymized) in the last 6 months of 2018 (control group) whose call details were captured from January 2019 to July 2019. Also, 135,696 women registered in the last 6 months of 2019 (treatment group) whose call details were captured from January 2020 to July 2020. For our analysis, we have considered only those women who have provided information on the following variables: phone ownership, enrollment channel, income range, age, and education.^16^ The response variables are call duration percentage and call duration range via classification in terms of green, amber, and red zones. We provide the variable descriptions in Table 1. We provide the summary statistics in Tables B1 and B2 in Appendix B.

**TABLE 1:** Variable description

Dependent variables	Definition and construction
Call duration percentage	If the call is picked up, this variable is calculated as (call duration/call length) × 100
Call duration range	Call duration percentage is divided into three zones – red (below 33%), amber (33–66%), and green (above 66%)

Independent variables	Definition and construction
COVID dummy	Coded as 1 if the date of the call starts from April and 0 otherwise
Treatment group	Women belong to the treatment group if they are called between January 2020 and July 2020. The variable is coded as 1 and 0 if they are called between January 2019 and July 2019
Husband phone owner	Coded as 1 if the ownership of the registered mobile phone is with the husband; 0 if the registered woman herself is the phone owner
Enrolled via community	Coded as 1 if a woman is enrolled in the program via a community channel and 0 if registered via a visit to a hospital
Lower income	Coded as 1 if the income of a woman lies in the bottom half of the sample and 0 otherwise

### 4.2 Did the COVID‐19 lockdown increase the duration of received calls?

In our empirical analyses to unpack the determinants of technological engagement, we tested whether the call was heard for a longer duration or not. This analysis directly examines Proposition 1 mentioned earlier. Our unit of observation is at the individual woman level. Our empirical model follows a difference‐in‐differences specification:

(1) $$y_{\mathrm{it}}=\beta_{0}+\beta_{1}{\text{TreatedGroup}}_{i}+\beta_{2}{\text{CovidDummy}}_{t}+\beta_{3}{\text{TreatedGroup}}_{i}\times {\text{CovidDummy}}_{t}+\theta_{i}+\delta_{t}+{ɛ}_{\mathrm{it}}$$

$\text{where}\;y_{\mathrm{it}}$ is used as call duration percentage (conditional on picking up the call) and call duration range (terciles of call duration percentage) in two separate models estimated. $y_{\mathrm{it}}$ is measured at the women–month level. The subscript *i* represents individual women. ${\text{TreatedGroup}}_{i}$ corresponds to whether women were registered in 2019 (1 for our treatment group) or registered in 2018 (0 for our control group). ${\text{CovidDummy}}_{i}$ equals one if the call was received after the COVID‐19 lockdown in India (April onward); it is zero otherwise. A similar design using the comparison of the treatment group and the control group across the year of COVID‐19 and the year before has been utilized by recent work (Gonzalez et al., 2020; Leslie et al., 2020; Ravindran & Shah, 2020).

Equation 1 represents our most saturated specification; in the baseline model, we estimate it without any fixed effects. Our coefficient of interest is $\beta_{3},$ which measures the percentage points change in call duration in the treatment cohort who listened to calls in 2020 compared to the control group who listened to calls in 2019 during the period of lockdown in India. Individual fixed effects ($\theta_{i})$ are included to control for time‐invariant heterogeneity across women. Month fixed effects ($\delta_{t}$) account for time‐varying common shocks. Standard errors are clustered at the individual women level in these specifications employing least square dummy variable regressions.

### 4.3 Which factors influence technology engagement?

Building on Proposition 2, we examine next how women grouped on phone ownership, enrollment channel, and monthly incomes exhibited heterogeneity in technology engagement due to the lockdown shock. We design a difference‐in‐differences model with the women registered in 2019, that is, those who heard calls from January 2020 to July 2020. The model captures the effect of COVID‐19 on three dimensions of woman‐level heterogeneity:

1. Husband being the phone owner (as opposed to women being the phone owner)
2. Women enrolled via a community channel (as opposed to enrolling at a hospital)
3. Women belonging to the lower‐income group (as opposed to the higher‐income group)

We estimate the following model:

(2) $$y_{\mathrm{it}}=\beta_{0}+\beta_{1}{\text{Group}}_{i}+\beta_{2}{\text{CovidDummy}}_{t}+\beta_{3}{\text{Group}}_{i}\times {\text{CovidDummy}}_{t}+\theta_{i}+\delta_{t}+{ɛ}_{\mathrm{it}}\;$$

where ${\text{Group}}_{i}$ varies as the husband being the phone owner (equals to 1 as opposed to 0 representing the woman being the phone owner), enrolled via community channel (equals to 1 as opposed to 0 representing direct enrollment via hospitals), and lower‐income group (equals to 1 as opposed to 0 representing a higher‐income group) for three different models. Individual fixed effects ($\theta_{i})$ are included to control for time‐invariant heterogeneity across women. Month fixed effects ($\delta_{t}$) account for time‐varying common shocks. Standard errors are clustered at the individual women level in all these specifications employing least square dummy variable regressions. Equation 2 represents the most saturated specification; in the baseline estimations, we would also wish to know the interaction coefficient without any fixed effects. Our coefficient of interest is $\beta_{3},$ which should measure the change in outcome variables due to the advent of COVID‐19 lockdown within the cohort of women who listened to calls in 2020.

In addition, we apply CEM in the specifications mentioned in Equations 1 and 2 to strengthen our identification strategy.

## 5 FINDINGS

### 5.1 Descriptive analysis

Tables B1 and B2 corroborate the aforementioned descriptive findings with summary statistics pre‐ and post‐March for women in 2019 (control group) and 2020 (treatment group). The mean values of the call duration percentage changed from 47.08 to 44.00 (6.54% decrease) from pre‐ to post‐March among the women in the control group (Table B2). For the treated group (Table B1), the same variable decreased from 45.46 to 43.68 (3.91% decrease). Mean figures indicate that though there is a decrease in call duration percentages in both groups, there is a 2.63 (6.54 − 3.91) percentage point *relative* increase in the call duration percentage in the treated group compared to the control group. This overall fall in engagement might seem counter to the key proposition that the COVID‐19 shock should lead to more engagements; we should also recognize that these are also non‐parametric summary statistics not controlling for heterogeneity, observed or unobserved. Also, intuitively, it may happen since we are considering women registered in the previous year, and generally, as time passes on from the time of registration, women tend to pick up calls less frequently. Comparing the relative magnitudes, we see that the fall in the treatment group is lesser in percentage terms. This difference captures the effect of the COVID‐19 shock.

A similar analysis for the call duration range in the green zone indicates a relative increase of 7.16 percentage points; the amber zone shows a relative decrease of 12.76 percentage points, and the red zone shows a relative decrease of 1.02 percentage points (Tables B1 and B2). Thus, a comparison of the mean values indicates a shift from calls being listened to in lower‐tercile zones (red and amber) to higher‐tercile zone (green). Overall, both call duration percentage and call duration range follow the same direction as hypothesized.

In Table 2, we compare raw mean differences in outcomes pre‐ and post‐pandemic for treatment and control groups. We analyze the difference using *t*‐statistics and corresponding *p*‐values. On observing the difference‐in‐differences estimate, we find a positive and significant increase in call duration percentage in all four models. Building on this, we conduct a more systematic empirical analysis in a regression framework in the next section.

**TABLE 2:** Descriptive estimates in the difference‐in‐differences framework

Duration percentage full sample	Pre	Post	Difference
Treatment	45.464	43.688	First difference = −1.776 (*t* = 24.256, *p* = 0.000)
Control	47.084	44.008	Second difference = −3.075 (*t* = 45.377, *p* = 0.000)
			Difference‐in‐differences = 1.299 (*t* = 13.03, *p* = 0.000)

Ownership	Pre	Post	Difference
Treatment	42.782	41.758	First difference = −1.023 (*t* = 7.412, *p* = 0.000)
Control	46.530	44.386	Second difference = −2.413 (*t* = 24.854, *p* = 0.000)
			Difference‐in‐differences = 1.119 (*t* = 6.84, *p* = 0.000)

Enrollment channel	Pre	Post	Difference
Treatment	42.266	41.691	First difference = −0.575 (*t* = 7.412, *p* = 0.000)
Control	51.141	47.002	Second difference = −4.139 (*t* = 34.269, *p* = 0.000)
			Difference‐in‐differences = 3.564 (*t* = 23.59, *p* = 0.000)

Income	Pre	Post	Difference
Treatment	43.993	42.435	First difference = −1.557 (*t* = 14.968, *p* = 0.000)
Control	46.878	44.891	Second difference = −1.987 (*t* = 24.854, *p* = 0.000)
			Difference‐in‐differences = 0.429 (*t* = 2.94, *p* = 0.003)

*Note*: The table represents the initial summary statistics in the difference‐in‐difference framework obtained from raw means. It includes the average call duration percentage obtained via four different classifications in data. The post‐period indicates months from April to July, and the pre‐period includes months from January to March.

### 5.2 Impact of the lockdown on technology engagement

Next, using Equation 1, we empirically evaluate Proposition 1 to measure the impact of COVID‐19 on technology engagement. Estimation results for the same are shown in Table 3. Column (1) is the baseline estimation without any fixed effects. In column (2), we introduce month fixed effects to account for seasonal heterogeneity. In column (3), along with month fixed effects, we introduce individual fixed effects to control for unobserved time‐invariant heterogeneity across women. In all estimations, standard errors are clustered at individual women levels.

**TABLE 3:** Change in call duration percentage in the treatment group during COVID‐19 lockdown

	(1)	(2)	(3)
Treatment group × COVID dummy	1.732***	1.695***	1.530***
	[0.087]	[0.087]	[0.088]
COVID dummy	−4.733***	−5.495***	−6.785***
	[0.065]	[0.085]	[0.089]
Treatment group	−1.065***	−1.058***	
	[0.126]	[0.126]	
Individual fixed effects	No	No	Yes
Month fixed effects	No	Yes	Yes
Observations	1,391,647	1,391,647	1,391,647
Number of women	252,145	252,145	252,145

*Notes*: The dependent variable in all columns is call duration percentage. Across model specifications, we see that the interaction term is positive and statistically significant. Thus, the call duration percentage increased significantly post‐COVID‐19. The time horizon is January 2020 to July 2020 (treatment group) and January 2019 to July 2019 (control group). The constant term is included but not reported. Robust clustered standard errors at the individual level are in parentheses. *p < .05, **p < .01, ***p < .001.

Results in Table 3 estimate how the COVID‐19 pandemic led to the women engaging with the calls for a longer duration. Columns (1)–(3) show significant positive coefficients indicating a relative increase in call duration percentage in the treatment group during the peak of COVID‐19 lockdown from April 2020 to July 2020. Depending on the specifications, we find a 1.53–1.73 percentage points increase in call duration percentage in the treatment group. This result supports Proposition 1, providing evidence of increased technology engagement.

Using Equation 2, we empirically evaluate Proposition 2 to disentangle the impact of COVID‐19 on the technology engagement arising from three demographic factors. Estimation for the same is shown in Table 4. In columns (1)–(3), we classify the treatment group of women based on phone ownership, enrollment channel, and income group, respectively. All specifications allow for individual and time fixed effects. In all estimations, standard errors are clustered at individual women levels.^17^

**TABLE 4:** Change in call duration percentage among different groups during COVID‐19 lockdown

	(1)	(2)	(3)
	Ownership	Enrolment channel	Income
Husband phone owner × COVID dummy	0.731***		
	[0.134]		
Enrolled via community × COVID dummy		2.756***	
		[0.123]	
Lower income × COVID dummy			0.494***
			[0.118]
COVID dummy	−5.755***	−7.306***	−5.833***
	[0.107]	[0.128]	[0.120]
Individual fixed effects	Yes	Yes	Yes
Month fixed effects	Yes	Yes	Yes
Observations	764,927	764,927	764,927
Number of women	135,696	135,696	135,696

*Notes*: The dependent variable in all columns is call duration percentage. In all specifications, we see that the interaction term is positive and statistically significant. In column (1), women are grouped based on mobile phone ownership. In column (2), women are grouped based on the enrollment channel. In column (3), the sample is grouped based on the monthly income. The time horizon is January 2020 to July 2020. The constant term is included but not reported. Robust clustered standard errors at the individual level are in parentheses. *p < .05, **p < .01, ***p < .001.

Results in Table 4 estimate how technology engagement is higher for certain classifications. Columns (1)–(3) show a significant positive coefficient indicating an increase in call duration percentage during the COVID‐19 lockdown from April 2020 to July 2020. Estimates in column (1) show a 0.73 percentage points increase in call duration for women whose husband (or a neighbor, in a few cases) was the phone owner compared to women who themselves owned the phone. Column (2) shows that the call duration is 2.75 percentage points higher if women are enrolled via community channels compared to the women enrolled in hospitals. In column (3), we find an increase in call duration of 0.49 percentage points for women who belong to the lower‐income group. Models (1) and (2) are in sync with Proposition 2. Model (3) shows that women in the poorer group are the ones who engage more with technology than richer women, as hypothesized before. An explanation is that conditional on purchasing the phone, information provision by ARMMAN is the only source of credible and useful information to these women leading to higher engagement, whereas the comparatively richer group of women may possibly have access to complementary sources for information. This explanation highlights the role of complementary information following the second mechanism under Proposition 2, indicating the importance of accessible information channels.

### 5.3 Stickiness in technology engagement

Next, we explore deeper to study how the women switched from low to high engagement with technology and, possibly more importantly, which group of women are less likely to switch, leading to stickiness. We adapt Equation 1 by changing the dependent variable to the call duration ranges captured by dummy variables and present the results in Table 5. To quantify the switch of women from low to high engagement, we divide the call duration as a percentage of the total call length into three zones of red, amber, and green following prior work (Clay‐Williams et al., 2019; Collins et al., 2014). Red indicates if the call gets heard for less than 33% of the total call length. Amber indicates the range of duration between 33% and 66%, and the green zone indicates if the call gets heard for more than 66% of the total call length.

**TABLE 5:** Change in call duration range during COVID‐19 lockdown

	(1)	(2)	(3)
	Green	Amber	Red
Treatment group × COVID dummy	0.036***	−0.031***	−0.005***
	[0.001]	[0.001]	[0.001]
COVID dummy	−0.103***	0.030***	0.073***
	[0.001]	[0.002]	[0.001]
Individual fixed effects	Yes	Yes	Yes
Month fixed effects	Yes	Yes	Yes
Observations	1,391,647	1,391,647	1,391,647
Number of women	252,145	252,145	252,145

*Notes*: The dependent variable in columns (1), (2), and (3) is the likelihood of the call duration range in the green, amber, and red zones, respectively. In model specification (1), we see that the interaction term is positive and statistically significant. In model specifications (2) and (3), we see it to be negative and significant. Thus, the likelihood of the call duration increased in the green zone and that in the amber and red zones decreased significantly post‐COVID‐19. The time horizon is January 2022 to July 2020 (treatment group) and January 2019 to July 2019 (control group). The constant term is included but not reported. Robust clustered standard errors at the individual level are in parentheses. *p < .05, **p < .01, ***p < .001.

The results in column (1) in Table 5 indicate a positive and significant increase of the magnitude of 3.60% in the green zone calls (calls heard for a higher duration) during the COVID‐19 lockdown among the women belonging to the treatment relative to the control group. At the same time, there is a decrease of 3.1% in the amber zone and 0.5% in red zone calls. Therefore, we conclude that the switch has occurred predominantly from amber zone to green zone. The women in the red zone remained sticky in their behavior and did not respond substantially.

Next, we use Equation 2 to analyze the effect on call duration range for different classifications of women in the treatment group. Estimation for the same is shown in Table 6. We observe that during the pandemic, the likelihood of calls in the green zone increased significantly for all three classifications, as shown in columns (1), (4), and (7). Similarly, the likelihood of call duration in the amber and red zones decreased significantly in all three groups split on ownership, enrollment channel, and income. A comparison of the estimates obtained in Tables 5 and 6 reveals a behavioral shift as women switch from the amber zone to green zone calls, whereas the women in the red zone remain sticky in response.

**TABLE 6:** Change in call duration range among different groups during COVID‐19 lockdown

	Ownership			Enrolment channel			Income		
	(1)	(2)	(3)	(4)	(5)	(6)	(7)	(8)	(9)
	Green	Amber	Red	Green	Amber	Red	Green	Amber	Red
Husband phone owner × COVID dummy	0.015***	−0.009***	−0.007***						
	[0.002]	[0.002]	[0.002]						
Enrolled via community × COVID dummy				0.045***	−0.015***	−0.030***			
				[0.002]	[0.002]	[0.002]			
Lower income × COVID dummy							0.011***	−0.006***	−0.005**
							[0.002]	[0.002]	[0.002]
COVID dummy	−0.070***	−0.006***	0.076***	−0.094***	0.001	0.092***	−0.072***	−0.005**	0.076***
	[0.002]	[0.002]	[0.002]	[0.002]	[0.002]	[0.002]	[0.002]	[0.002]	[0.002]
Individual fixed effects	Yes	Yes	Yes	Yes	Yes	Yes	Yes	Yes	Yes
Month fixed effects	Yes	Yes	Yes	Yes	Yes	Yes	Yes	Yes	Yes
Observations	764,927	764,927	764,927	764,927	764,927	764,927	764,927	764,927	764,927
Number of women	135,696	135,696	135,696	135,696	135,696	135,696	135,696	135,696	135,696

*Notes*: The dependent variable in columns (1), (4), and (7) is the likelihood of the call duration range in the green zone. The dependent variable in columns (2), (5), and (8) is the likelihood of the call duration range in the amber zone. The dependent variable in columns (3), (6), and (9) is the likelihood of the call duration range in the red zone. The likelihood of the call duration in the green zone increased, and that in the amber and red zones decreased significantly post‐COVID‐19 in all three models. The time horizon is January 2020 to July 2020. The constant term is included but not reported. Robust clustered standard errors at the individual level are in parentheses. *p < .05, **p < .01, ***p < .001.

### 5.4 Discussion on pre‐trends

We check for the existence of pre‐trends following Angrist and Pischke (2008). The econometric specification given in Equation 3 is used to generate coefficient plots using an event study design.

(3) $$\text{Call}\;{\text{Duration Percentage}}_{\mathrm{it}}=\beta_{0}+\beta_{1}{\text{TreatedGroup}}_{i}+\beta_{2}{\text{Month}}_{t}+\sum \beta_{t}\;\left({\text{TreatedGroup}}_{i}\times {\text{Month}}_{t}\right)+\theta_{i}+{ɛ}_{\mathrm{it}}$$

**FIGURE 2:**
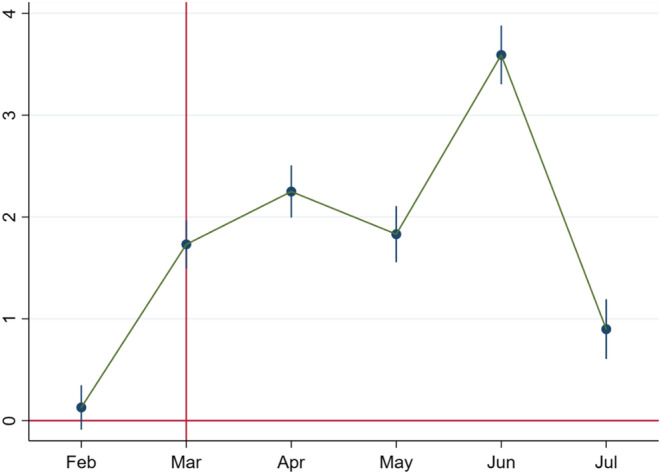
Coefficient plot for call duration percentage in the full sample. Based on Equation 3, we plot the coefficients over time. The *x*‐axis denotes January 2020 to July 2020 (treatment) and January 2019 to July 2019 (control). The coefficient estimates for the call duration percentage do not have any pre‐trends in February. The effect starts in March, which can be attributed to the fact that March contained a week of the post‐lockdown period (the lockdown started on March 24, 2020). The shift in the coefficients for the call duration percentage from zero to positive and significant values post‐COVID‐19 is evident and fully consistent with the baseline difference‐in‐differences results

${\text{Month}}_{t}$ varies from February to July. (January, the first period, is taken as the base.) Individual fixed effects ($\theta_{i})$ are included to control for time‐invariant heterogeneity across women. Standard errors are clustered at the individual women level in all these specifications employing least square dummy variable regressions. We look for insignificant coefficients in the pre‐trend period of February to signify the absence of pre‐trends. In Figure 2, we plot coefficients from Equation 3, taking January 2020 to July 2020 as the treatment group and January 2019 to July 2019 as the control group. We find that there is no significant difference in the treatment and control groups till the end of February. The coefficient is positive in March, which can be possibly attributed to the fact that March contained a week of the post‐lockdown period (the lockdown started on March 24, 2020).

**FIGURE 3:**
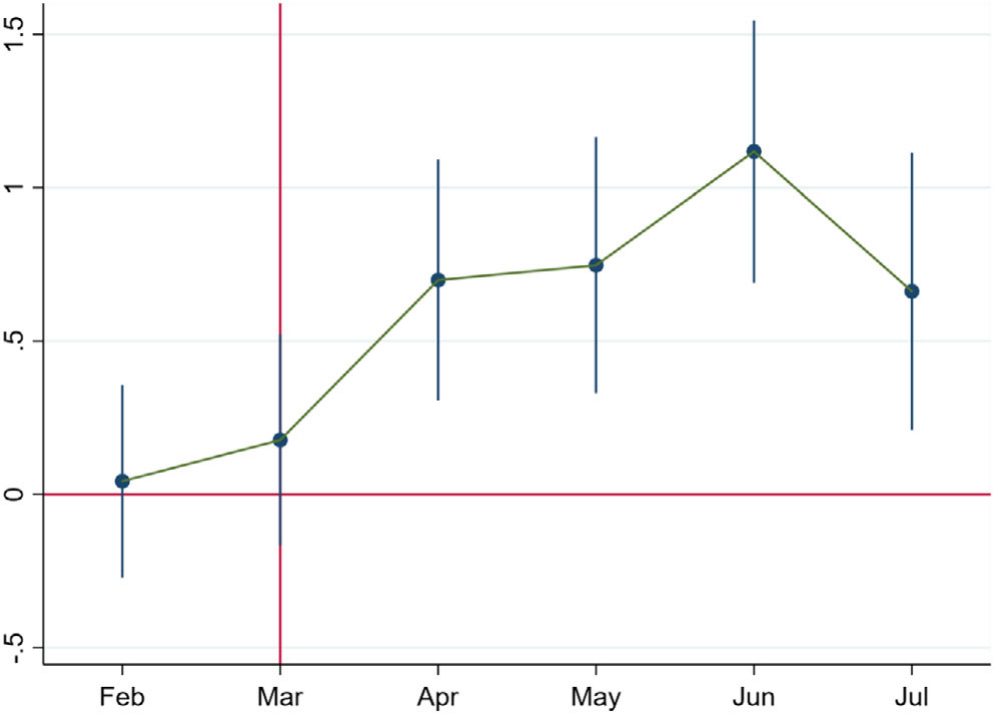
Coefficient plot for the call duration percentage based on phone ownership. Based on Equation 3, we plot the coefficients over time. The *x*‐axis denotes January 2020 to July 2020. The coefficient estimates for the call duration percentage do not have any pre‐trends. The shift in the coefficients for the call duration percentage from zero to positive and significant values post‐COVID‐19 is evident and fully consistent with the baseline difference‐in‐differences results

**FIGURE 4:**
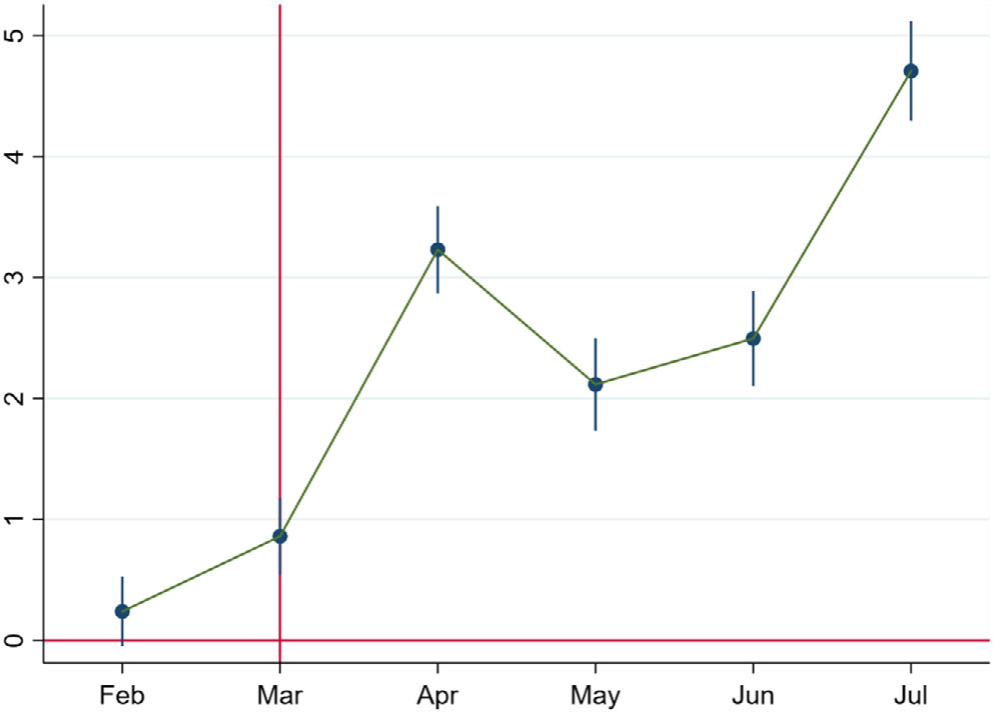
Coefficient plot for the call duration percentage based on the enrollment channel. Based on Equation 3, we plot the coefficients over time. The *x*‐axis denotes January 2020 to July 2020. The coefficient estimates for the call duration percentage do not have any pre‐trends in February. The effect starts in March, which can be attributed to the post‐lockdown period in March (the lockdown started on March 24, 2020). The shift in the coefficients for the call duration percentage from zero to positive and significant values post‐COVID‐19 is evident and fully consistent with the baseline difference‐in‐differences results

**FIGURE 5:**
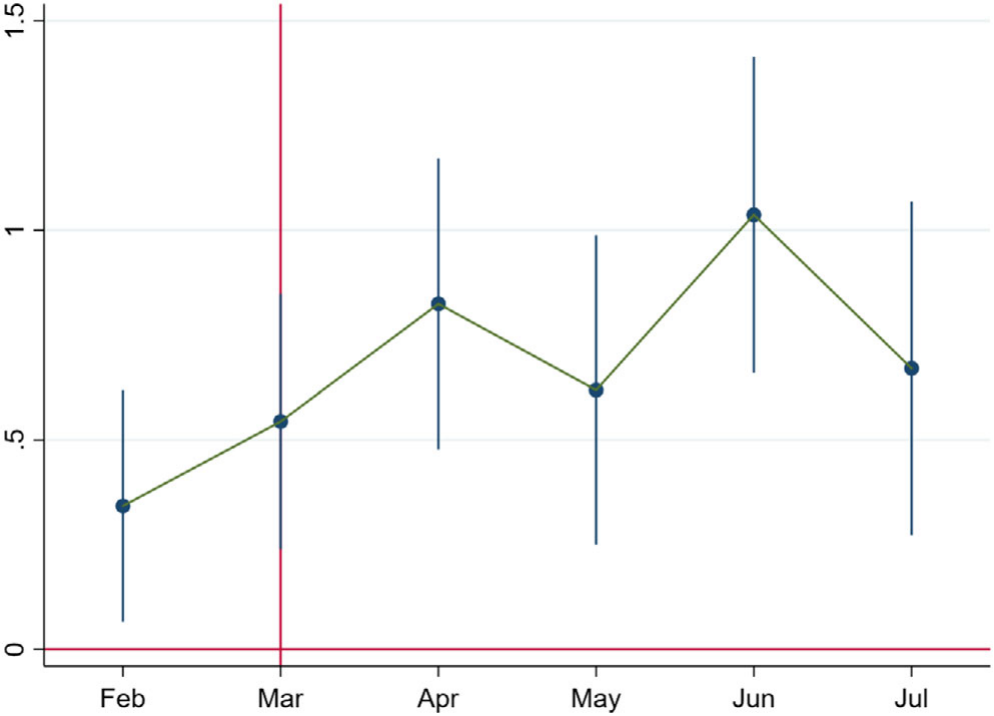
Coefficient plot for the call duration percentage based on income. Based on Equation 3, we plot the coefficients over time. The *x*‐axis denotes January 2020 to July 2020. The existence of pre‐trends cannot be denied. The shift in the coefficients for the call duration percentage from zero to positive and significant values post‐COVID‐19 is evident and fully consistent with the baseline difference‐in‐differences results

In Figures 3, 4, 5, we focus only on women who listened to calls from January 2020 to July 2020. Following the specification in Equation 2, we classify the treatment group based on phone ownership, enrollment channel, and monthly income. In Figures 3, 4, 5, we generate the coefficient plots for these three variables in the same way as Equation 3. In Figure 3, we find the nonexistence of pre‐trends, along with a sharp rise in call duration percentage from April 2020. Figure 4 follows the same pattern as Figure 3, along with a sharp rise from April 2020. In Figure 5, we cannot rule out the existence of pre‐trends. However, a shift in the magnitudes of the estimates from April is quite evident. Taken together, these results show that ownership, enrollment channels, and income influence the extent of technology engagement.

## 6 ROBUSTNESS CHECKS

### 6.1 CEM estimates

For robustness check, we employ the CEM strategy (Iacus et al., 2012). CEM is a method for estimating causal effects by reducing imbalance in covariates between treated and control groups (Blackwell et al., 2009). CEM is easy to use and requires fewer assumptions. These advantages of the CEM method have led to frequent usage of this method in multiple recent studies (Wang & Overby, 2021; Wang et al., 2021; Azoulay et al., 2019) as a means for robustness test for difference‐in‐differences estimates where the choice of control groups may bias the resulting estimates.

In our case, the variables of interest are call duration percentages and the corresponding ownership, enrollment channel, and income variables of the women. We match the treatment and control groups of women based on their reported age and education. Estimates generated by CEM on coarsened data are shown in Table 7. The results are in line with our baseline estimates strengthening our inference.

**TABLE 7:** Coarsened exact matching (CEM) estimate

	(1)	(2)	(3)	(4)
	Full sample	Ownership	Enrolment channel	Income
Treatment group × COVID dummy	1.705***			
	[0.090]			
Husband phone owner × COVID dummy		0.758***		
		[0.138]		
Enrolled via community × COVID dummy			2.851***	
			[0.125]	
Lower income × COVID dummy				0.498***
				[0.123]
COVID dummy	−6.976***	−5.941***	−7.442***	−5.939***
	[0.092]	[0.119]	[0.131]	[0.127]
Individual fixed effects	Yes	Yes	Yes	Yes
Month fixed effects	Yes	Yes	Yes	Yes
Observations	1,391,680	764,585	764,845	764,768
Number of women	252,150	135,633	135,678	135,668

*Notes*: The dependent variable in all columns is call duration percentage. In model specifications (1) to (4), the interaction term is positive and statistically significant. Across model specifications, we see that coefficients obtained after CEM follow the same sign and significance as baseline results. The time horizon is January 2020 to July 2020 (treatment group) and January 2019 to July 2019 (control group) for specification (1) and January 2020 to July 2020 for specifications (2), (3), and (4). The constant term is included but not reported. Robust clustered standard errors at the individual level are in parentheses. *p < .05, **p < .01, ***p < .001.

### 6.2 Placebo test with a 2017–2018 sample

To add to our identification strategy, we also conduct a placebo test wherein we replicate all our estimations with a timeline that is advanced by 7 months (compared to the length of the timeline in the main specification), excluding overlap with the main timeline. We categorize the women registered with ARMMAN in 2018 as the treatment group and women registered in 2017 as the control group and generate the baseline result as shown in Table 8 (column 1). For demographic heterogeneity, we use the data of women registered in 2018 in the same way as we did in our main estimation strategy. In all cases, we find the interaction coefficients to be either insignificant or in the opposite direction of the baseline estimates. While in an ideal world, all estimates should have been insignificant as well, we note two things. One, the outreach for ARMMAN was increasing and it did not stabilize in the first few years of existence, which coincided with this timeline. Therefore, some unstable estimates are expected to be there. More importantly, we find that the lockdown effect manifests in the opposite direction, indicating that the gross impact was larger.

**TABLE 8:** Placebo test

	(1)	(2)	(3)	(4)
	Full sample	Ownership	Enrolment channel	Income
Treatment group × Placebo_Dummy	0.182			
	[0.125]			
Husband phone owner × Placebo_Dummy		−1.776***		
		[0.213]		
Enrolled via community × Placebo_Dummy			0.142	
			[0.204]	
Low income × Placebo_Dummy				−0.694***
				[0.206]
Placebo_Dummy	1.652***	2.660***	1.958***	2.324***
	[0.162]	[0.251]	[0.260]	[0.252]
Individual fixed effects	Yes	Yes	Yes	Yes
Month fixed effects	Yes	Yes	Yes	Yes
Observations	1,429,851	511,375	511,375	511,375
Number of women	455,751	147,962	147,962	147,962

*Notes*: The dependent variable in all columns is call duration percentage. In model specifications (1) to (4), the interaction term is insignificant or negative. The time horizon is June 2018 to December 2018 (treatment group) and June 2017 to December 2017 (control group) for specification (1) and June 2018 to December 2018 for specifications (2), (3), and (4). The treatment month is September. The constant term is included but not reported. Robust clustered standard errors at the individual level are in parentheses. *p < .05, **p < .01, ***p < .001.

## 7 SUMMARY AND DISCUSSION

Mobile devices with digitization have become a powerful policy measure for information dissemination due to widespread availability and low cost, even in developing countries. However, while the supply side of technology engagement is well studied, the demand side remains relatively less studied and understood. There is recent work on what kinds of nudges make people adopt technologies (Breza et al. 2021; Ghose et al. 2021; Siddiqui et al. 2021; Banerjee et al. 2020; Ghose et al. 2017). However, there is a gap in understanding how various factors of economic and non‐economic nature influence the intensive margins of engagement.

In this paper, we address this question in the Indian context with a unique organizational data set from enrolled users of ARMMAN, an Indian nonprofit organization that sends timely informational calls via mobile phones to less‐privileged pregnant women around the Mumbai metropolitan area, courtesy their mMitra program. Listening to the voice messages may involve the pecuniary and non‐pecuniary costs of various forms for the target group of women. We posit that technology engagement is a function of ownership, accessibility of different channels of information, and income effect. Using a difference‐in‐differences setup, we first establish that compared to the previous year, there has been a sizeable increase in technology engagement of pregnant women during the COVID‐19 lockdown that India imposed. Our estimate indicates an increase of 1.53 percentage points in the call duration percentage in the treatment group compared with the control group. Given the increase in technology engagement, we examine the factors influencing the choice of engagement with technology. For this purpose, we focus on the 2020 cohort to explore the three factors possibly influencing technology engagement. We find that ownership, enrollment channel, and monthly income play a significantly important role in inducing technology engagement.

While the level of engagement increases, a follow‐up question arises as to whether the average effect is the same for all women. We complement this analysis with call duration ranges to characterize women's switch from one range to another. This analysis shows that women who were not listening to the calls before the exogenous shock respond minimally post the shock. However, the women who had spent even a moderate amount of time listening to the calls before the shock increased their engagement significantly, driving the overall increase in engagement. All our results are robust to CEM estimates.

Our study contributes to the intersection of three different strands of the literature on technology engagement, m‐Health, and the effects of economic and demographic factors influencing technology engagement behavior. Our results can potentially inform policymakers in this rapidly expanding domain of m‐Health, especially in an economy with a lack of information and associated institutional setup. Understanding technology engagement at an extensive margin would require knowledge of sociopolitical factors involved at that time. With restricted data availability and context specificity, in this paper, we wish to contribute to the literature on technology engagement only at the intensive margin.

More broadly, we attend to calls for a deeper and nuanced understanding of how digitization affects the *information‐rich* and *information‐poor*, given the renewed impetus on technology in the world recovering from the pandemic. This is especially important as digitization figures more prominently in the UN SDGs. Finally, in contrast to many recent studies showing an adverse impact of the pandemic on women, our findings show that, though unintended, the pandemic increased technological engagement for pregnant women, which eventually could have improved their health. This is a lesson structurally worth considering for public health measures tomorrow when the world deals with not just pandemics of the future but also with gender‐induced distortions and inefficiencies in an unequal world.

Our study is not without limitations. First, we note that ARMMAN has a geographically concentrated base, with a majority of enrollments from the urban slums of Mumbai. This limits demographic variation in the data. However, since the main mechanism is based on trade‐offs in time, income, and channels in accessing information, we consider our results to be relatively robust toward other unobserved demographic characteristics. In addition, in our estimation, we incorporate individual fixed effects to account for unobserved individual heterogeneity.

In conclusion, we acknowledge the scope of future work building on our results. While the current literature focuses on the effects of interventions from many dimensions, one should explore the degree of persistence of this behavioral shift. Future work should also complement our findings to conduct detailed text analytics of the nature of engagement with technology for the populace beyond the duration of the engagement. While we were restricted in terms of data access, it would be interesting to examine if technology engagement of the nature we study translates into positive effects on health outcomes of individuals, quantifying welfare implications of technology engagement in the society with the rapid proliferation of communication technology. Also, while the paucity of data prevented us from examining it, future research should examine heterogeneities in technology engagement for women who had given birth before compared to women who were pregnant for the first time. Finally, our work demonstrates the need for a time‐use study of how women allocate their time during the day in developing economy contexts like India during COVID‐19.

## CONFLICT OF INTEREST

The findings and opinions expressed in this paper are those of the authors and are not in any way reflective of ARMMAN's. The paper was written in complete independence, and ARMMAN, as an institution, did not weigh in on the analysis of the data or the drafting of the report.

## Figures and Tables

**FIGURE C1:**
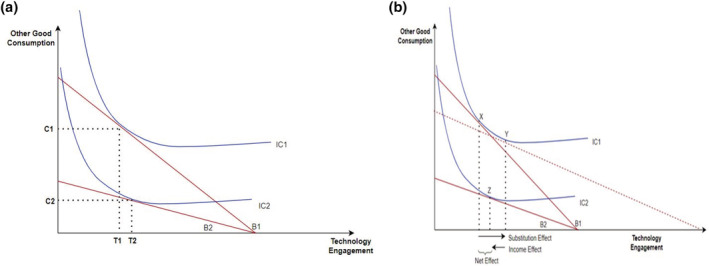
Variation in technology engagement due to the COVID‐19‐induced lockdown. The budget curve in Panel A rotates counterclockwise due to the wage drop, reflecting the contraction of the budget set. The welfare‐maximizing choice of technology engagement T2 in the lockdown period is larger than the prelockdown value T1. Panel B provides a decomposition of the same change via income and substitution effects

## Data Availability

Data sharing is not applicable to this article as no new data were created or analyzed in this study.

## Endnotes

^1^
We would like to present our clarification on how we measure call duration. First, the call duration recorded by NGO ARMMAN starts only after the pregnant woman picks up the call. The measurement of call duration happens only when the person listening picks up the call pushed by NGO ARMMAN through the mMitra program and does not include the caller tune duration that may precede it. Thus, the caller tune of COVID‐19 message is not part of the call duration calculations for our intervention that we examine in this paper. We thank an anonymous referee for raising this issue helping us to explicitly clarify how we measure call duration in the paper.

^2^
The lockdown ensuing from COVID‐19 in India was one of the strictest lockdowns globally (see https://www.theguardian.com/commentisfree/2020/jul/04/india-lockdowns-cases-rising)

^3^
We have imputed a categorical variable across the available income ranges to estimate the mean income, as we do not see the exact value of the income. For each range, we consider the middle value of the income range to represent the average income. Rupee to US dollar exchange rate was around 73 in March 2020. Therefore, the mean income of the lower‐income group—Rs. 6,500—would be equivalent of approximately USD89.

^4^
UNGIS: United Nations Group on the Information Society.

^5^
The 2030 Agenda for Sustainable Development, adopted by all UN Member States in 2015, provides a shared blueprint for peace and prosperity for people and the planet, now and into the future. At its heart are the 17 SDGs, which are an urgent call for action by all countries—developed and developing—in a global partnership—https://sdgs.un.org/goals

^6^
See “Opinion | What we see in the shameful trends on U.S. maternal health,” *The New York Times* (nytimes.com), last accessed June 2, 2022; we thank Rema Padman for pointing this out to us.

^7^
ASHA stands for Accredited Social Health Activists in the Indian public health ecosystem; see https://en.wikipedia.org/wiki/Accredited_Social_Health_Activist

^8^
ImTecho stands for Innovative Mobile‐phone Technology for Community Health Operations.

^9^
MNCH stands for Mother, Newborn and Child Health.

^10^

https://armman.org

^11^
BabyCenter LLC (based in San Francisco) is an international organization that provides service to improve maternal health and works closely with nonprofits, NGOs, and governments to help women achieve safe motherhood.

^12^

https://www.mohfw.gov.in

^13^

https://www.mygov.in/COVID-19

^14^

https://www.cbsnews.com/news/india-coronavirus-covid19-poor-hit-hardest-lockdown-extended-narendra-modi-today-2020-04-14/

^15^
“The pandemic has worsened India's poverty crisis,” *The Indian Express*, July 14, 2021. https://indianexpress.com/article/opinion/the-pandemic-has-worsened-indias-poverty-crisis-7394367/

^16^
Our sample consists of all women with complete data. In case any of the three dimensions of information are not found for a given individual, we ignore that particular woman and her call details.

^17^
We have replicated the baseline estimations without clustered standard errors, and they show the same sign and significance as baseline (see Table B3).

## APPENDIX B

**TABLE B1:** Summary statistics for treatment group with call details from January 2020 to July 2020 before and after April

January 2020 to March 2020	*N*	Mean	SD	Min.	Max.
Call duration (%)	4,02,809	45.464	33.686	0	100
Green	4,02,809	0.358	0.479	0	1
Amber	4,02,809	0.224	0.417	0	1
Red	4,02,809	0.416	0.493	0	1
Husband phone owner	3,54,512	0.255	0.436	0	1
Enrolled via community	3,54,512	0.640	0.479	0	1
Low income	3,54,512	0.536	0.498	0	1

April 2020 to July 2020	*N*	Mean	SD	Min.	Max.
Call duration (%)	4,54,448	43.688	33.966	0	100
Green	4,54,448	0.332	0.470	0	1
Amber	4,54,448	0.228	0.419	0	1
Red	4,54,448	0.439	0.496	0	1
Husband phone owner	4,10,415	0.255	0.436	0	1
Enrolled via community	4,10,415	0.630	0.482	0	1
Low income	4,10,415	0.536	0.498	0	1

**TABLE B2:** Summary statistics for the control group with call details from January 2019 to July 2019 before and after April

January 19 to March 19	*N*	Mean	SD	Min.	Max.
Call duration (%)	3,96,560	47.084	34.408	0	100
Green	3,96,560	0.388	0.487	0	1
Amber	3,96,560	0.213	0.409	0	1
Red	3,96,560	0.397	0.489	0	1

April 19 to July 19	*N*	Mean	SD	Min.	Max.
Call duration (%)	6,46,344	44.008	33.100	0	100
Green	6,46,344	0.332	0.471	0	1
Amber	6,46,344	0.244	0.429	0	1
Red	6,46,344	0.423	0.494	0	1

**TABLE B3:** Baseline results without clustered standard errors

	(1)	(2)	(3)	(4)
	Full sample	Ownership	Enrolment channel	Income
Treatment group × COVID dummy	1.530***			
	[0.072]			
Husband phone owner × COVID dummy		0.731***		
		[0.110]		
Enrolled via community × COVID dummy			2.756***	
			[0.099]	
Low income × COVID dummy				0.494***
				[0.096]
COVID dummy	−6.785***	−5.755***	−7.306***	−5.833***
	[0.077]	[0.093]	[0.109]	[0.102]
Individual fixed effects	Yes	Yes	Yes	Yes
Month fixed effects	Yes	Yes	Yes	Yes
Observations	1,391,647	764,927	764,927	764,927
Number of women	252,145	135,696	135,696	135,696

*Note*: The dependent variable in all columns is the call duration percentage. In model specifications (1) to (4), the interaction term is positive and statistically significant. Across model specifications, we see that coefficients obtained without clustering follow the same sign and significance as baseline results. The time horizon is January 2020 to July 2020 (treatment group) and January 2019 to July 2019 (control group) for specification (1) and January 2020 to July 2020 for specifications (2), (3), and (4). The constant term is included but not reported.

## APPENDIX C

### A CONCEPTUAL FRAMEWORK ANCHORING OUR EMPIRICAL FINDINGS

The current COVID‐19 has caused damaging changes in labor supply and leisure choices through many channels (Fadlon & Nielsen, 2021). Building on our empirical findings, we next delineate a theoretical model to explain and illustrate how a negative shock in the form of the lockdown ensuing from the COVID‐19 pandemic may affect technology engagement behavior.

We assume a generic representative woman with utility following constant elasticity of substitution (CES) utility function (Zabalza, 1983) along with budget constraints, as shown in the following equation:

$$\begin{array}{cc} \mathrm{Max}. & \;U\left(t,c\right)={\left(\propto t^{\rho }+\left(1-\propto \right)c^{\rho }\right)}^{\frac{1}{\rho }}\\ \;\text{subject to} & P_{c}\;c+w\;t=w \end{array}$$

where *t* represents technology engagement, *c* represents the consumption of all other goods, $P_{c}$ is the price of consumption of all other goods, and *w* is the wage rate. The parameters ∝ and ρ represent the relative weightage of *t* and *c* and the degree of substitution, respectively.

The budget constraint is motivated by the standard microeconomic labor–leisure choice model (Aguiar & Hurst, 2016; Clerc et al., 2012; Heckman, 1974). We assume the amount of time available in a day to be 1, out of which *t* shall be spent on consuming technology and (1 − *t*) is spent on earning wage rate *w*. The money spent on all other consumption should be less than or equal to the money earned through wages, that is,

$$P_{c}\;c\leq w\;\left(1-t\right).$$

This constraint can be rewritten as (the inequality sign will be redundant as for a maximize of utility, no residual wage will be left):

$$P_{c}\;c+w\;t=w.$$

The representative woman maximizes her utility subject to the budget constraint. To obtain an equilibrium value of technology engagement $t^{*}$, we write the Lagrangian as

$$L={\left(\propto t^{\rho }+\left(1-\propto \right)c^{\rho }\right)}^{1/\rho }+\lambda \;\left(P_{c}\;c+w\;t-w\right)$$

where λ is the Lagrange multiplier. By differentiating the Lagrangian with respect to the arguments, we get

(5)
$$\partial L/\partial c=1/\rho {\left(\propto t^{\rho }+\left(1-\propto \right)c^{\rho }\right)}^{1/\rho -1}\;\left(1-\alpha \right)\rho \;c^{\rho -1}+\lambda \;P_{c}=0$$

and

(6)
$$\partial L/\partial t=1/\rho {\left(\propto t^{\rho }+\left(1-\propto \right)c^{\rho }\right)}^{1/\rho -1}\;\alpha \rho \;t^{\rho -1}+\lambda \;w=0$$

From Equations 5 and 6, we get

$$\left(\left(1-\alpha \right)/\alpha \right){\left(c/t\right)}^{\rho -1}=P_{c}/w$$

which can be rewritten as

$$c^{*}=t\;{\left(\alpha /\left(1-\alpha \right)\right)}^{1/\left(\rho -1\right)}\left(P_{c}\;\right)/w^{1/\left(\rho -1\right)}.$$

The elasticity of substitution $r\;\text{is given}\;\mathrm{by}\;1/\left(1-\rho \right)$. As −∞ < ρ< 1, the range of *r* is given by 0 < r <∞. Utilizing *r*, we can rewrite the optimal consumption of all other goods as

$$\;c^{*}=t{\left(\left(1-\alpha \right)/\alpha \right)}^{r}{\left(w/\left(P_{c}\;\right)\right)}^{r}.$$

Substituting the value of *c** in Equation 4, we get

$$P_{c}\;t\;{\left(\left(1-\alpha \right)/\alpha \right)}^{r}{\left(w/\left(P_{c}\;\beta \right)\right)}^{r}+t\;w=w,$$

which we can solve for the expression of the optimal level of technology engagement:

$$t^{*}=\frac{w\;}{P_{c}\;{\left(\left(1-\alpha \right)/\alpha \right)}^{r}{\left(w/\left(P_{c}\;\right)\right)}^{r}+w}.$$

$$t^{*}={\left[1+{P_{c}}^{1-r}A^{-r}w^{r-1}\right]}^{-1}$$

where *r* is the elasticity of substitution $\text{given}\;\mathrm{by}\;1/\left(1-\rho \right).$ (As −∞ < ρ< 1, 0 < *r* <∞.) Also, A = $\alpha /\left(1-\alpha \right).$ (As 0<α < 1, 0 < A < ∞.).

To observe the effect of the negative wage shock on technology engagement (*t*), we differentiate $t^{*}$ with respect to *w* and we get

$$\frac{\partial t^{*}}{\partial w}=\frac{\left(-1\right)\left(r-1\right){P_{c}}^{1-r}A^{-r}w^{r-2}}{{\left[1+{P_{c}}^{1-r}A^{-r}w^{r-1}\right]}^{2}}\;$$

Since $P_{c},A,r,w$ are all positive values, and the denominator, being a squared term, is always positive. Therefore, $\partial t^{*}/\partial w<0\;\text{if}\;r>1$. Thus, a negative wage shock shall increase technology engagement if the substitution effect dominates the income effect. We explain this in Figure C1.

We plot the indifference curves IC1 and IC2 subjected to change in budget constraint from B1 to B2 owing to the negative wage shock due to the pandemic. Panel A shows how in equilibrium, the shock caused an increase in technology engagement while there was a drop in the consumption of other goods. Panel B shows that the negative pandemic shock causes both substitution and income effects, but the substitution effect dominates the income effect, causing a net positive increase in technology engagement. Thus, a negative wage shock caused by the pandemic would lead to an increase in technology engagement when the substitution effect dominates the income effect.
